# Unraveling the Angiogenic Puzzle: Pre-Treatment sVEGFR1 and sVEGFR2 Levels as Promising Prognostic Indicators in Early-Stage Breast Cancer Patients

**DOI:** 10.3390/ijms241713508

**Published:** 2023-08-31

**Authors:** Elżbieta Zarychta, Kornel Bielawski, Katarzyna Wrzeszcz, Piotr Rhone, Barbara Ruszkowska-Ciastek

**Affiliations:** 1 Department of Pathophysiology, Faculty of Pharmacy, Nicolaus Copernicus University, Collegium Medicum, 9 Curie Sklodowska Street, 85-094 Bydgoszcz, Poland; kornel-bielawski@wp.pl (K.B.); katarzyna.wrzeszcz@abs.umk.pl (K.W.); ruszkowska.basia@gmail.com (B.R.-C.); 2 Invicta Fertility Clinic, 6 Zlota Street, 00-019 Warsaw, Poland; 3 Clinical Ward of Breast Cancer and Reconstructive Surgery, Oncology Centre Prof. F. Łukaszczyk Memorial Hospital, 2 Romanowska Street, 85-796 Bydgoszcz, Poland; rhonep@co.bydgoszcz.pl

**Keywords:** angiognesis, VEGF-A, breast cancer treatment, breast cancer metastasis, breast cancer prognosis

## Abstract

Despite the advancements in breast cancer (BrC) diagnosis and treatment, a considerable proportion of patients with early-stage disease still experience local recurrence or metastasis. This study aimed to assess the levels of specific angiogenic parameters in the EDTA plasma of BrC patients before and after treatment and to explore their clinical and prognostic significance. The levels of vascular endothelial growth factor A (VEGF-A), soluble form of vascular endothelial growth factor receptor type 1 (sVEGFR1), and soluble form of vascular endothelial growth factor receptor type 2 (sVEGFR2) were measured in 84 early BrC patients, both prior to surgery and within a median time of nine months post-treatment. Prognostic significance was evaluated using Kaplan-Meier survival and Cox regression analyses. Linear regression models were employed to examine the independent impact of selected angiogenic factors on DFS in breast cancer patients. The results of uni- and multivariate analyses indicated that a pre-treatment concentration of sVEGFR1 above 30.99 pg/mL was associated with improved disease-free survival (DFS) (*p* < 0.0001 for both analyses), while a pre-treatment concentration of sVEGFR2 above 9475.67 pg/mL was associated with an increased risk of BrC relapse (*p* < 0.0001 for both analyses). Additionally, a post-treatment concentration of sVEGFR2 above 7361.71 pg/mL was associated with better overall survival (OS) based on the Kaplan-Meier survival analysis (*p* = 0.0141). Furthermore, linear regression models revealed a significant inverse association between pre-treatment levels of sVEGFR1 and the risk of relapse (standardized β −0.2578, *p* = 0.0499) and a significant positive association of VEGF-A levels with the risk of recurrence (standardized β 0.2958, *p* = 0.0308). In conclusion, the findings suggest that both pre- and post-treatment levels of sVEGFR1 and sVEGFR2 may hold promise as potential prognostic markers for BrC patients.

## 1. Introduction

Breast cancer (BrC), being the most prevalent invasive cancer in females worldwide, remains a significant public health concern. This disease exhibits heterogeneity, characterized by variations in the histological type, grade, and expression of immunohistochemical markers, which subsequently guide the determination of optimal treatment strategies [1,2]. While the introduction of targeted therapies in recent decades has contributed to improved survival rates, the effectiveness of treatment largely depends on the stage of the disease. Early-stage BrC can be completely curable; however, metastatic dissemination still carries a grim prognosis. Despite advancements in BrC therapy, preventing disease recurrence and metastasis poses a formidable challenge for oncologists, given that approximately 20–30% of patients with early-stage BrC will experience disease spread [3]. Hence, understanding the underlying molecular mechanisms responsible for BrC progression and systemic dissemination is of paramount importance in the quest for developing novel treatment approaches [4,5].

In the management of early-stage BrC, standard therapeutic approaches typically involve surgical interventions such as breast-conserving surgery (BCS) or mastectomy, accompanied by axillary staging. Subsequently, radiotherapy and adjuvant systemic treatment are considered. The selection of systemic therapy is contingent upon the molecular subtype of BrC. Endocrine therapy is recommended for tumors that express estrogen or progesterone receptors, commonly referred to as luminal BrCs. This therapeutic strategy encompasses the use of either tamoxifen or an aromatase inhibitor. Chemotherapy is indicated for triple-negative BrC, HER2-positive BrC, or luminal-like HER2-negative BrC with a high-risk profile. Patients with tumors overexpressing the HER2 protein are treated with immune therapy, specifically utilizing trastuzumab [6].

BrC, like other solid tumors, necessitates the development of its own blood supply, a process known as angiogenesis, to surpass a size of 1–2 mm in diameter [7]. Angiogenesis not only plays a critical role in tumor growth but also contributes significantly to the formation of metastases in BrC [8,9]. Vascular endothelial growth factor (VEGF) is a primary angiogenic factor in BrC, and its levels in the bloodstream correlate with the disease stage [10,11,12]. The biological effects of VEGF-A (vascular endothelial growth factor A) are mediated by binding to two homologous receptors, namely vascular endothelial growth factor receptor 1 (VEGFR1 or Flt-1) and vascular endothelial growth factor receptor 2 (VEGFR2 or KDR). These receptors are predominantly expressed in endothelial cells (ECs), although they can also be found in non-endothelial cells [13].

Numerous antiangiogenic therapies have been developed for the treatment of BrC; however, their efficacy has been limited despite promising outcomes in preclinical studies [14,15]. Angiogenesis inhibitors can function by impeding the activity of pro-angiogenic factors, such as Bevacizumab (an anti-VEGF monoclonal antibody), or by targeting their receptors, as exemplified by Ramucirumab (an anti-VEGFR2 monoclonal antibody). These agents aim to disrupt the supply of nutrients and oxygen to the tumor [9]. Additionally, they can act as tyrosine kinase inhibitors, including Sorafenib, Sunitinib, Vandetanib, Axitinib, Pazopanib, and Cediranib, which inhibit the kinase domain of the tyrosine kinase receptor, leading to the inhibition of receptor activation and downstream signaling pathways. Despite their use, the effectiveness of antiangiogenic drugs remains modest, often accompanied by subsequent therapy resistance. Furthermore, these agents are associated with a range of side effects, such as hypertension, hemorrhagic complications, and an increased risk of thromboembolism, ultimately limiting their clinical utility [9,16].

Despite significant advancements in the field of BrC therapy, there remain significant gaps in our understanding, particularly concerning the intricate mechanisms underlying angiogenesis and vasculogenesis in the context of BrC development and systemic dissemination. Thus, the focus of this study was to comprehensively examine the levels of angiogenic parameters both before and after treatment and to determine their association with the specific treatment modalities employed. Furthermore, we sought to assess the prognostic value of these parameters in patients diagnosed with BrC. By elucidating these intricate relationships, we aim to contribute valuable insights into the prognostic potential and therapeutic implications of angiogenesis-related mechanisms in BrC.

## 2. Results

### 2.1. Patient-Specific Data

The study cohort consisted of 84 individuals diagnosed with BrC who underwent primary surgical intervention followed by subsequent treatments such as radiation therapy and systemic therapy, including chemotherapy, immunotherapy, or endocrine therapy. The demographic and clinical characteristics of the study population are summarized in Table 1. 

### 2.2. Assessment of Angiogenic Parameters Regarding the Type of Treatment

The concentrations of VEGF-A, sVEGFR1, and sVEGFR2 were assessed before and after treatment, categorized by the type of treatment received by the patients. The results are presented in Table 2 (VEGF-A), Table 3 (sVEGFR1), and Table 4 (sVEGFR2). In the group of patients who underwent breast-conserving surgery (BCS) followed by radiotherapy, the median post-treatment VEGF-A concentration was significantly higher compared with pre-treatment levels (106.9 vs. 65.85 pg/mL; *p* = 0.0028). However, no significant difference in post-treatment VEGF-A levels was observed in the mastectomy group (without subsequent radiotherapy) (55.02 vs. 53.17 pg/mL; *p* = 0.7893).

Regarding sVEGFR1, post-treatment concentrations were significantly higher compared with pre-treatment levels in patients treated surgically, irrespective of the type of surgery, radiation therapy, or the implementation of chemotherapy or endocrine therapy. 

In contrast to that, post-treatment concentrations of sVEGFR2 were significantly lower compared with pre-treatment levels in our cohort, irrespective of the type of surgical treatment or radiation therapy (BCS followed by radiotherapy (7397.15 vs. 9275.74 pg/mL; *p* < 0.0001; mastectomy (7165.78 vs. 10,140.8 pg/mL; *p* = 0.0033, respectively). Post-treatment concentrations of sVEGFR2 were also significantly lower in patients who underwent endocrine treatment with tamoxifen (7458.0 vs. 9868.01 pg/mL; *p* = 0.0018) or an aromatase inhibitor (7400 vs. 9581.91 pg/mL; *p* = 0.0059). Furthermore, post-treatment sVEGFR2 levels were lower independently from administration or type of chemotherapy; however, only in patients treated with anthracyclines or in patients not receiving chemotherapy were those differences statistically significant (7327.4 vs. 8751.85 pg/mL; *p* = 0.0176; 7422.15 vs. 9990.63 pg/mL; *p* = 0.0002, respectively).

### 2.3. Survival Analysis Regarding Angiogenic Parameters

During the follow-up period, which had a median duration of 74 months, we observed a total of ten deaths (11.9%) and four cases of disease relapse (5%) among the patients included in our study. To evaluate the impact of pre- and post-treatment concentrations of VEGF-A, sVEGFR1, and sVEGFR2 on OS and DFS, we categorized the patients into two groups based on the median cut-off points for each angiogenic parameter. Specifically, one group comprised patients with levels below the respective cut-off value, while the other group consisted of patients with levels above the cut-off value. The median cut-off points for VEGF-A were 64.49 pg/mL (pre-treatment) and 100.11 pg/mL (post-treatment); for sVEGFR1, they were 30.99 pg/mL (pre-treatment) and 338.95 pg/mL (post-treatment); and for sVEGFR2, they were 9475.67 pg/mL (pre-treatment) and 7361.71 pg/mL (post-treatment).

**Figure 1:**
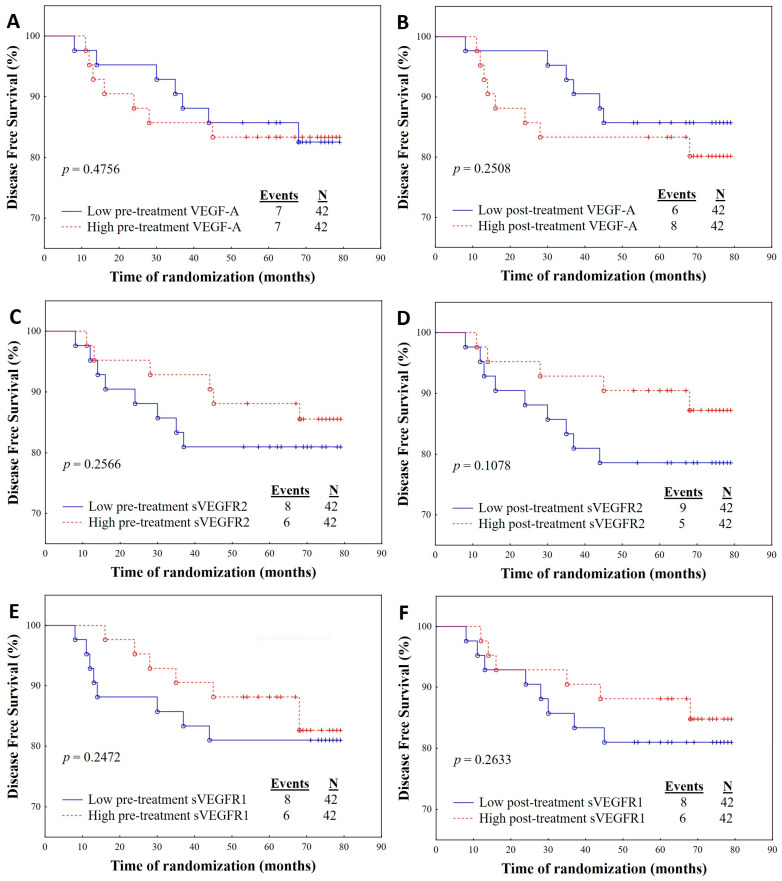

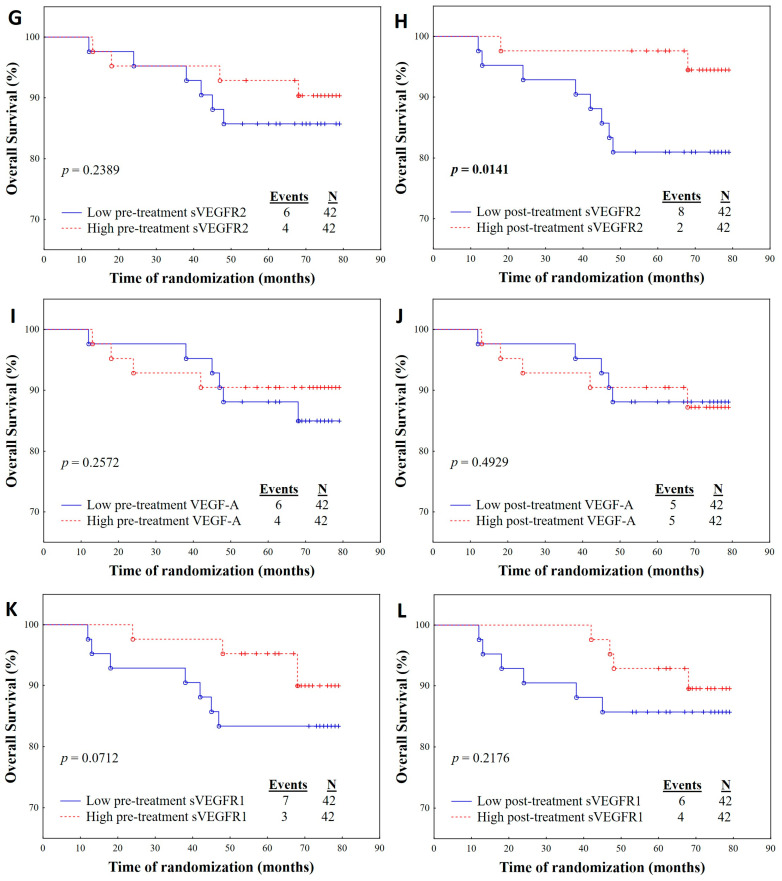
Kaplan Meier curves displaying the estimated survival probability for different groups of patients, who were treated for breast cancer. (**A**–**F**) depict Disease-Free Survival (DFS) outcome predicated upon the stratification of individuals according to their levels of angiogenic biomarkers before and subsequent to treatment administration. (**G**–**L**) depict the trend of Overall Survival (OS) outcome contingent upon the classification of patients in accordance with their angiogenic marker concentrations pre- and post-intervention. Patients with a post-treatment sVEGFR2 concentration above 7361.71 pg/mL exhibited significantly better OS compared with those with a post-treatment sVEGFR2 concentration below this threshold (*p* = 0.0141).

The Kaplan-Meier analysis revealed that patients with a post-treatment sVEGFR2 concentration above 7361.71 pg/mL exhibited significantly better OS compared with those with a post-treatment sVEGFR2 concentration below this threshold (*p* = 0.0141) (Figure 1H). Specifically, out of the 42 patients with post-treatment sVEGFR2 concentrations above 7361.71 pg/mL, only two events (4.76%) occurred, whereas in the group of 42 patients with concentrations below this threshold, eight events (19.05%) took place. 

### 2.4. Association of OS and DFS with Angiogenic Parameters

Uni- and multivariate Cox regression analyses (Table 5 and Table 6) were conducted to identify the clinicopathological variables that exert an influence on prognosis. The results from both uni- and multivariate analyses revealed that a pre-treatment concentration of sVEGFR1 exceeding 30.99 pg/mL was significantly associated with an extended DFS period (HR = 0.3104, 95%CI 0.1893–0.5075, *p* < 0.0001 for univariate analysis; HR = 0.2170, 95%CI 0.1204–0.3912, *p* < 0.0001 for multivariate analysis). Furthermore, a pre-treatment concentration of sVEGFR2 surpassing 9475.67 pg/mL was significantly associated with an elevated risk of BrC relapse (HR = 2.9558, 95%CI = 1.8071–4.8344, *p* < 0.0001 for univariate analysis; HR = 4.2506, 95%CI = 2.3292–7.7571, *p* < 0.0001 for multivariate analysis).

### 2.5. Association of Angiogenic Parameters with DFS in Linear Regression Models

In order to assess the independent impact of specific angiogenesis factors on DFS, we constructed four linear regression models. The associations between pre- and post-treatment concentrations of VEGF-A, sVEGFR1, and sVEGFR2 with DFS were examined using multiple regression analyses, as presented in Table 7. In Model 3, which was adjusted for age, BMI, parity, menopausal status, and smoking status, the results demonstrated a significant negative association between the pre-treatment concentration of sVEGFR1 and the risk of BrC relapse (standardized β −0.2578, *p* = 0.0499). Additionally, in Model 4, adjusted for BMI, parity, menopausal status, smoking status, disease stage, tumor diameter, intrinsic type, histological type, and nodal involvement, higher pre-treatment levels of VEGF-A were associated with an increased risk of BrC recurrence (standardized β 0.2958, *p* = 0.0308).

## 3. Discussion

Malignant tumors with a rapid growth rate necessitate the development of new blood vessels to meet their increased demands for nutrients and oxygen. Within the microenvironment of BrC, both angiogenic and non-angiogenic vascularization pathways can be observed [9]. The process of angiogenesis, primarily mediated by VEGF-A, plays a crucial role in promoting the formation of new blood vessels. VEGF-A belongs to the VEGF family of cytokines, which also includes VEGF-B, VEGF-C, VEGF-D, VEGF-E, and placental growth factor (PlGF) [17]. VEGF-A exerts its angiogenic effects by directly stimulating endothelial cells (ECs), leading to their proliferation and the subsequent formation of new blood vessels. Additionally, VEGF-A indirectly influences angiogenesis by inhibiting apoptosis in ECs and modulating the activity of enzymes responsible for extracellular matrix degradation [18]. Moreover, VEGF-A enhances vascular permeability, induces chemotaxis, and upregulates the expression of plasminogen activators and collagenases [18,19].

In this study, we aimed to investigate the prognostic significance of angiogenic parameters, specifically VEGF-A and its receptors, in early-stage BrC patients by evaluating their pre- and post-treatment levels. Previous research has established that patients with primary BrC exhibit significantly higher levels of VEGF-A, sVEGFR1, and sVEGFR2 compared with healthy individuals [20]. However, the impact of treatment on these angiogenic parameters remains poorly understood.

Regarding VEGF-A levels, we observed a significant increase only in patients who underwent radiotherapy, while other types of treatment did not influence their levels. This finding can be explained by the fact that radiotherapy alters tumor perfusion, and the effect depends on the treatment dosage and setup. Low-dose fractionated radiotherapy may promote tumor perfusion and oxygenation by inducing angiogenesis through the expression of VEGF-A by tumor cells or cells within the tumor microenvironment. On the other hand, higher-dose irradiation (above 10 Gy) leads to acute vascular damage and cell death. Consequently, vascular regression and collapse occur, leading to decreased tumor perfusion and hypoxia. Interestingly, this may trigger a vascular rebound effect, inducing vasculogenesis through endothelial progenitor cells from other parts of the body [21,22]. Furthermore, our findings regarding VEGF-A levels before and after chemotherapy align with those of Karsten et al., who reported that chemotherapy did not affect VEGF-A levels in BrC patients [23].

Regarding the levels of sVEGFR1 and sVEGFR2, we observed significant changes after various treatments. Levels of sVEGFR1 increased significantly after surgical treatment, with or without radiotherapy, as well as after chemotherapy or endocrine therapy. Conversely, levels of sVEGFR2 decreased significantly after surgical treatment, with or without radiotherapy, chemotherapy (specifically in patients treated with anthracycline-based chemotherapy), and endocrine therapy (either tamoxifen or aromatase inhibitor). To the best of our knowledge, no studies have compared plasma levels of sVEGFR1 or sVEGFR2 in patients primarily treated with surgery and adjuvant endocrine therapy or chemotherapy. However, a study by Banerjee et al. demonstrated a significant increase in sVEGFR1 in BrC patients treated with neoadjuvant anastrozole but not with tamoxifen [24]. Nevertheless, due to differences in study design, direct comparisons with our research are not feasible.

Furthermore, we investigated whether plasma levels of VEGF-A, sVEGFR1, and sVEGFR2 could serve as biomarkers for the risk of disease relapse or progression in invasive BrC patients. Using the univariate Cox regression model, we found that pre-treatment plasma levels of sVEGFR1 exceeding 30.99 pg/mL were associated with improved DFS, while pre-treatment plasma levels of sVEGFR2 exceeding 9475.67 pg/mL were associated with shorter DFS. These observations were confirmed through multivariate analysis. We hypothesize that elevated pre-treatment levels of sVEGFR1 and decreased pre-treatment levels of sVEGFR2 may serve as determinants of a better prognosis in BrC patients. Importantly, the introduction of BrC treatment, such as surgery, radiotherapy, or systemic therapy, led to changes in the angiogenic profiles of patients, specifically an increase in sVEGFR1 levels and a decrease in sVEGFR2 levels, which is a remarkable finding.

In cancer development and progression, VEGF-A exerts its effects by binding to its receptors, VEGFR1 and VEGFR2. Activation of VEGFR2 stimulates angiogenesis by increasing endothelial cell (EC) proliferation [13,25,26]. It also enhances vascular permeability, promotes cell migration, and stimulates the release of von Willebrand factor (vWF) by activated ECs [13,25,26,27]. VEGFR1, although not involved in EC proliferation, plays a role in EC differentiation and migration. It exhibits a higher affinity for VEGF-A than VEGFR2 [13] and acts as a counterbalance by preventing the binding of VEGF-A to VEGFR2, thus inhibiting the proangiogenic signals mediated by VEGF-A [13,25,27,28]. The soluble form of VEGFR1, generated through alternative splicing of the VEGFR1 gene, lacks the transmembrane and intracellular domains but still maintains a high affinity for VEGF-A. It is speculated to function by reducing or modulating the actions of VEGF-A [13,25,29]. In a study conducted by Toi et al., a soluble VEGFR1 concentration surpassing the intra-tumoral VEGF-A concentration by 10-fold was associated with a favorable prognosis in BrC patients [29]. Another possible mechanism of VEGF-A regulation by sVEGFR1 involves the formation of inactive heterodimeric receptor complexes with VEGF receptors, primarily VEGFR2, thereby impeding the angiogenic potential of VEGF-A [30]. These actions of sVEGFR1 may explain the observed relationship between increased sVEGFR1 levels and a favorable prognosis in BrC patients in our study. The hypothesis regarding the functionality of sVEGFR1 as a trap for VEGF-A appears to be supported by empirical and clinical evidence. Specifically, the presence of elevated pre-treatment levels of sVEGFR1 in patients afflicted with rectal carcinoma, glioblastoma, triple-negative BrC, or hepatocellular carcinoma indicates a diminished response to treatment with bevacizumab-based therapy. This is due to the limited likelihood of significant biological effects resulting from the introduction of external anti-VEGF agents in patients exhibiting high levels of sVEGFR1 (already acting as an anti-VEGF agent). Furthermore, it is worth noting that heightened concentrations of sVEGFR1 have also been correlated with a reduced occurrence of adverse effects in patients diagnosed with these malignancies [31].

Similarly, a soluble form of VEGFR2 (sVEGFR2) has been identified in humans, presumably secreted by ECs [32]. While sVEGFR1 acts as a trap receptor for VEGF-A, thereby suppressing its activity, sVEGFR2 does not directly suppress VEGF-A-mediated activity. However, it may function as an inhibitor of VEGF-C-induced lymphangiogenesis, which also plays a crucial role in cancer development [32]. Additionally, VEGF-A may be responsible for the down-regulation of membrane-bound VEGFR2 on ECs, resulting in a correlated decrease in plasma levels of sVEGFR2. This decrease could indicate low levels of sVEGFR2 as a marker of increased circulating VEGF-A and tumor growth [33]. This hypothesis is consistent with our findings that higher post-treatment levels of sVEGFR2 are associated with improved OS, according to Kaplan-Meier analysis.

Investigating the levels of VEGF-A, sVEGFR1, and sVEGFR2 before and after different treatments revealed intriguing patterns, which point toward their potential roles as indicators of treatment response and prognosis. The findings obtained from longitudinal analysis have the potential to enhance risk stratification and treatment decisions for individual patients. This approach has several implications for future research. Firstly, it highlights the need for comprehensive, long-term studies that follow patients throughout their treatment journey to capture the nuanced changes in angiogenic profiles over time. Secondly, it underscores the importance of understanding the mechanisms underlying treatment-induced changes in angiogenic factors, shedding light on potential avenues for therapeutic interventions targeting angiogenesis. Finally, it emphasizes the value of personalized medicine, where patient-specific molecular profiles can guide treatment choices and predict outcomes.

### Limitations and Strengths of the Study

The findings of this study should be interpreted with caution, as there are several limitations. The study has a relatively small sample size of 84 patients, which limits statistical power and generalizability. Conducting the study at a single center may restrict the applicability of the findings to other populations or healthcare settings. Additionally, the study’s inclusion of patients treated at a specific clinical ward may introduce selection bias, as these patients may not fully represent the broader population of BrC patients. The absence of a control group hinders the ability to assess the observed changes in angiogenic parameters. The study’s geographical focus may limit the generalizability of the findings to diverse BrC populations worldwide, as regional factors such as genetic variations or healthcare practices can influence outcomes. Lastly, there may be unmeasured confounding factors, such as socioeconomic status, lifestyle factors, or genetic variations, which were not accounted for in the analysis, potentially impacting the results. The strengths of this study include its prospective design, well-defined patient selection criteria, use of standardized treatment protocols, comprehensive data collection, and long-term follow-up. Conducting the study prospectively allows for the collection of data in real-time, reducing the risk of recall bias. The well-defined cohort of early-stage BrC patients treated at a single center enhances internal validity and enables more accurate analysis. Following standardized treatment protocols based on national guidelines ensures consistency and minimizes confounding factors. The comprehensive data collection, encompassing various clinical and demographic variables, facilitates thorough analysis and exploration of potential associations. The long-term follow-up, with a median duration of 74 months, provides valuable information on disease-free survival and overall survival, enabling an assessment of the prognostic efficacy of the measured angiogenic parameters.

## 4. Materials and Methods

### 4.1. Study Design

The present study is a prospective single-center observational investigation that obtained data from a cohort of patients diagnosed with invasive BrC. The participants were exclusively treated at the Clinical Ward of Breast Cancer and Reconstructive Surgery, located within the Oncology Center at Prof. F. Łukaszczyk Memorial Hospital in Bydgoszcz, Poland. The data collection period spanned from November 2015 to June 2017. 

**Figure 2:**
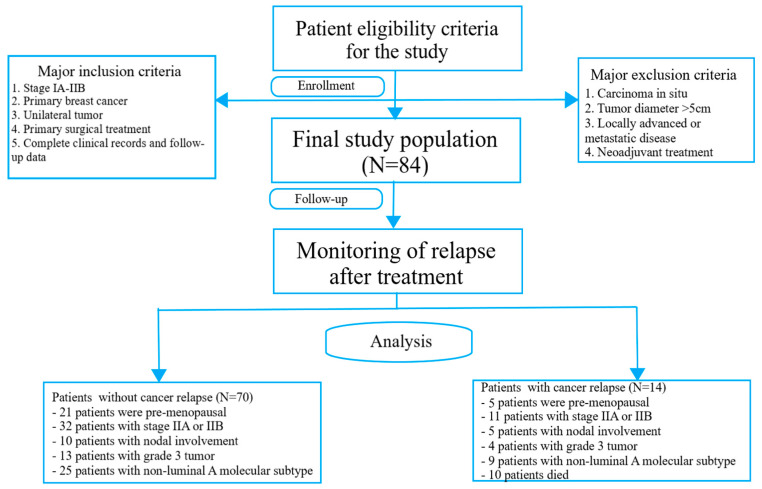
Patient selection protocol.

The cohort consisted of 84 patients diagnosed with early-stage BrC, specifically stages IA to IIB. The primary treatment approach involved surgical intervention, followed by adjunctive therapies such as radiation therapy and/or systemic treatments including chemotherapy, immunotherapy, or endocrine therapy. Histological confirmation of the diagnosis was obtained for each patient in accordance with prevailing pathology guidelines. Further post-surgical evaluations were performed to determine important clinical factors, including tumor size, lymph node involvement, and tumor stage. The TNM staging system, as outlined by the American Joint Committee on Cancer (AJCC) in its 7th edition, was employed to accurately classify the disease stage at the time of initial diagnosis [34]. The process of patient selection is depicted in Figure 2.

The health-related data of the study participants was acquired through comprehensive subject interviews and thorough physical examinations conducted prior to their enrollment in the study. A range of pertinent medical information was meticulously collected, including reproductive history, menopausal status, menopausal hormone therapy, the presence of comorbidities, current medication usage, smoking status, previous occurrences of breast or other types of cancer, details of the treatment regimen received, and the subjects’ body mass index (BMI). Menopause was defined as the absence of menstruation for a duration of 12 consecutive months in women aged 40 years or older. The BMI, a measure of body weight relative to height, was calculated by dividing the patient’s weight in kilograms by the square of their height in meters, as recorded during the initial visit.

### 4.2. Adjuvant Treatment

All patients enrolled in this study received treatment in accordance with the national breast cancer guidelines, specifically following the treatment patterns outlined by the National Comprehensive Cancer Network (NCCN) Guidelines for Practice. A multidisciplinary team, comprised of a clinical oncologist, radiologist, surgeon, and radiation therapist, recommended adjuvant therapy for eligible patients after the initial surgical intervention, which involved either breast-conserving surgery (BCS) or mastectomy.

The adjuvant therapy options included radiation therapy, chemotherapy, endocrine therapy, or immune therapy, and the specific treatment modalities were determined based on the individual patient’s condition and the assessment of the risk of disease recurrence. 

Radiotherapy was administered to patients who underwent BCS, had positive nodal status, or had narrow surgical margins. The treatment protocol involved the delivery of a median dose of 45 gray (Gy) in 17–20 fractions over a period of 4–6 weeks, utilizing tangential photon fields. For patients with N1 status, additional radiation was applied to the supraclavicular, infraclavicular, and axillary nodes using an anterior field that was matched to the tangential fields. In cases where there was a high risk of disease relapse, a sequential boost of 10 Gy was administered in five fractions directly to the initial tumor bed using a direct electron field.

Systemic treatment encompassed chemotherapy, endocrine therapy, immune therapy, or a combination thereof, and it was initiated within 2–4 weeks after surgery, based on the tumor type and the assessment of recurrence risk. Patients with a luminal A subtype of BrC typically received endocrine therapy alone, while those with a luminal B HER2(-) subtype received a combination of endocrine therapy and chemotherapy. In cases of luminal B HER2(+) BrC, immunotherapy in the form of trastuzumab, a humanized anti-HER2 monoclonal antibody, was also administered. For non-luminal tumor treatment, options included chemotherapy and immune therapy. Patients with triple-negative BrCs were offered chemotherapy as a standalone treatment. Adjuvant chemotherapy, either anthracycline-based or without anthracycline, was typically administered over 4–6 cycles. Endocrine therapy, which depended on menopausal status, involved the use of tamoxifen in premenopausal women or aromatase inhibitors (letrozole, anastrozole, or exemestane) in postmenopausal women, and in some cases, a combination of both, for a minimum of five years. 

All relevant data pertaining to adjuvant treatment was diligently collected and documented for analysis in this study.

### 4.3. Patient Outcomes

The cohort of 84 patients diagnosed with early-stage BrC was included in the study and follow-up. Throughout a median follow-up period of 74 months, a total of 14 events were recorded, comprising four cases of disease relapse and ten deaths. Patient follow-up was conducted via telephone calls or clinical visits, which continued until either 30th January 2022 or the date of their demise, ensuring comprehensive monitoring and data collection.

Disease-free survival (DFS) and overall survival (OS) were determined by calculating the time elapsed from the day of surgery to the occurrence of the first event, which could be disease relapse or death. The median DFS was found to be 70.5 months (interquartile range [IQR]: 62–77 months). Similarly, the median OS was observed to be 72.5 months (IQR 63–77 months). 

### 4.4. Methods

#### 4.4.1. Blood Sampling and Angiogenic Parameters Evaluation 

A total of two venous blood samples were obtained from all subjects in this study. The first blood withdrawal occurred on the day prior to the primary breast surgery, while the second sample was collected at a median time of nine months after the surgery. The blood specimens were collected using tubes containing 1.8 mg of ethylenediaminetetraacetic acid (EDTA), which served as an anticoagulant. These samples were used to measure the concentrations of key angiogenic parameters, namely vascular endothelial growth factor-A (VEGF-A), the soluble form of vascular endothelial growth factor receptor type 1 (sVEGFR1), and the soluble form of vascular endothelial growth factor receptor type 2 (sVEGFR2).

Following collection, the blood samples underwent standard processing procedures—they were subjected to centrifugation at a speed of 3000 times the force of gravity (3000× *g*) for a duration of 15 min. Subsequently, the plasma obtained from the centrifuged samples was carefully stored at a temperature of −80 °C until further analysis. To determine the concentrations of the angiogenic parameters of interest, namely VEGF-A, sVEGFR1, and sVEGFR2, enzyme-linked immunosorbent assay (ELISA) kits were employed. Specifically, the VEGF-A concentrations were measured using the VEGF Immunoassay Test (Quantikine, cat. number: DVE00) from R&D Systems, USA. Similarly, the concentrations of sVEGFR1 were determined using the sVEGFR1/Flt-1 Immunoassay (Quantikine, cat. number: DVR100B), and the concentrations of sVEGFR2 were determined using the sVEGFR2/KDR Immunoassay (Quantikine, cat. number: DVR200), both also from R&D Systems, USA. The reaction mixture containing the samples and appropriate reagents was added to a 96-well plate. The ELISA kits utilized in this study had respective mean detectable EDTA plasma values of approximately 61.0 pg/mL for VEGF-A, 80.0 pg/mL for sVEGFR1/Flt-1, and 9577 pg/mL for sVEGFR2/KDR. These values represent the lower limits of detection for each respective parameter.

#### 4.4.2. Immunohistochemistry

The intrinsic molecular subtypes of BrC were determined using immunohistochemical (IHC) analysis of specific markers, including the estrogen receptor (ER), progesterone receptor (PR), human epidermal growth factor receptor 2 (HER2), and Ki67 proliferative marker. These markers were utilized to classify BrC into distinct subtypes, namely luminal A, luminal B HER2-positive, luminal B HER2-negative, non-luminal HER2-positive, and triple-negative. Tumors exhibiting at least 1% of tumor cell staining for ER or PR were considered to have positive receptor status. The evaluation of HER2 status was performed by a pathologist, who assessed the staining intensity of HER2 using the IHC scoring system. Based on the intensity of staining, the HER2 status was categorized as positive (IHC score = 3+), negative (IHC score = 0 or 1+), or equivocal (inconclusive IHC score = 2+). If results were equivocal, testing was performed using in situ hybridization in accordance with the ASCO/CAP guidelines [35]. This classification provided valuable information regarding the HER2 expression in the BrC samples, aiding in the determination of the intrinsic subtype of the cancer.

### 4.5. Statistical Analysis

The primary focus of this study was to investigate the survival outcomes, specifically DFS and OS, in patients with stage IA–IIB BrC based on the levels of VEGF-A, sVEGFR1, and sVEGFR2 before and after treatment. The secondary objectives were to explore the relationship between surgical and adjuvant treatments and the concentrations of VEGF-A, sVEGFR1, and sVEGFR2. Additionally, the study aimed to assess the risk of disease relapse, considering various factors such as age, BMI, parity, menopausal status, smoking status, tumor stage, tumor diameter, intrinsic and histological type of BrC, and nodal involvement.

The collected data were examined for normality using the Shapiro-Wilk test, and non-normally distributed variables were presented as medians and interquartile ranges (IQRs). To assess differences between groups, the Mann-Whitney U test was employed. Survival curves were constructed using the Kaplan-Meier method and compared using the log-rank test. Statistical significance was set at a *p*-value of less than 0.05.

To determine the impact of various variables on DFS and OS, both univariate and multivariate analyses were conducted using the Cox proportional hazards regression test. The results were expressed as hazard ratios (HRs) with corresponding 95% confidence intervals (CIs). Additionally, multiple linear regression models were developed to examine the independent effects of specific angiogenesis factors on DFS.

All statistical analyses were performed using Statistica v.13.1 software (StatSoft, Cracow, Poland) to ensure accuracy and reliability in the interpretation of the data.

### 4.6. Ethics Approval

Prior to their enrollment, each subject provided informed consent and demonstrated their understanding of the study’s purpose and procedures in writing. The ethical guidelines outlined in the Code of Ethics of the World Medical Association, as documented in the Declaration of Helsinki published in the British Medical Journal on 18 July 1964, were strictly adhered to. The study protocols were additionally approved by the local bioethical committee under permission number KB 547/2015.

## 5. Conclusions

This study provides evidence supporting the potential prognostic value of pre-treatment levels of sVEGFR1 and sVEGFR2 in BrC patients, indicating their association with DFS. Specifically, higher pre-treatment levels of sVEGFR1 and lower pre-treatment levels of sVEGFR2 were found to be associated with improved DFS outcomes. Additionally, our findings demonstrate significant associations between various BrC treatments, such as surgery, radiotherapy, chemotherapy, and endocrine therapy, and changes in angiogenic parameters. Notably, higher post-treatment levels of sVEGFR2 were associated with improved OS, according to the Kaplan-Meier analysis. These results underscore the potential importance of angiogenic parameters as markers for predicting treatment response and patient outcomes in BrC. However, further investigation is warranted to elucidate the underlying mechanisms driving changes in angiogenic parameters and their implications for BrC treatment response and patient prognosis.

## Figures and Tables

**Table 1:** Baseline characteristics of the study group.

Patient Characteristics	Number of Patients (%)
Age [years]	
<50	23 (27)
≥50	61 (73)
Menopausal status	
Premenopausal	26 (31)
Postmenopausal	58 (69)
BMI [kg/m^2^]	
18.5–24.9	42 (50)
25–29.9	28 (33)
≥30	14 (17)
Parity	
Nulliparous	8 (10)
Parous	76 (90)
Cigarette smoking	
Smoker	20 (24)
Non-smoker	64 (76)
Stage	
IA	42 (50)
IB	0 (0)
IIA	37 (44)
IIB	5 (6)
Tumor diameter	
T1a	5 (6)
T1b	8 (10)
T1c	44 (52)
T2	27 (32)
Nodal involvement	
Negative	64 (76)
Positive	20 (24)
Intrinsic type	
Luminal A	51 (61)
Luminal B HER2(-)	16 (19)
Luminal B HER2(+)	5 (6)
Non-luminal HER2(+)	3 (3)
Triple-negative	9 (11)
Histological type	
Invasive ductal	73 (87)
Invasive lobular	11 (13)
Grading	
1	4 (5)
2	64 (76)
3	16 (19)
Disease recurrence	
Yes	4
No	80
DFS (months)	
Median (IQR)	70.5 (62–77)
Died during follow-up	
Yes	10
No	74
OS (months)	
Median (IQR)	72.5 (63–77)

BMI—body mass index; HER2—human epidermal growth factor receptor 2; T1a—tumor size >1 mm but ≤5 mm; T1b—tumor size >5 mm but ≤10 mm; T1c—tumor size >10 mm but ≤20 mm; T2—tumor size >20 mm but ≤50 mm; DFS—disease free survival; OS—overall survival; IQR—interquartile range.

**Table 2:** Levels of VEGF-A in breast cancer patients before and after treatment.

Feature/ Number of Patients (%)	Pre-Treatment VEGF-A Concentration (pg/mL)	Post-Treatment VEGF-A Concentration (pg/mL)	
	Median (Q1–Q3)	Median (Q1–Q3)	*p*-Values
BCS + Radiotherapy n = 70 (83%)	65.85 (44.51–116.35)	106.90 (58.14–161.70)	**0.0028**
Mastectomy n = 14 (17%)	53.17 (38.41–102.82)	55.02 (34.78–110.20)	0.7893
Chemotherapy			
Anthracycline n = 30 (36%)	52.005 (38.41–76.83)	83.33 (37.31–145.20)	0.2012
Non-anthracycline n = 8 (9%)	62.69 (43.43–108.60)	80.58 (61.44–115.76)	0.2888
No n= 46 (55%)	82.63 (39.67–126.40)	114.05 (56.43–161.70)	0.1048
Endocrine therapy			
Tamoxifen n = 41 (49%)	56.32 (39.67–111.09)	98.43 (49.77–144.40)	0.2115
Inhibitor aromatase n = 19 (23%)	66.83 (32.47–118.34)	105.5 (56.43–163.10)	0.0665
Tamoxifen and inhibitor aromatase n = 7 (8%)	74.12 (38.41–126.93)	52.61 (28.98–161.70)	1.0000
Other type n = 3 (4%)	64.87 (37.45–81.45)	68.14 (52.25–96.96)	0.2482
No n = 14 (17%)	80.84 (49.70–115.39)	130.15 (81.75–156.70)	0.4227

VEGF-A—vascular endothelial growth factor A; BCS—breast conserving surgery; significant differences are denoted by bold *p*-values.

**Table 3:** Levels of sVEGFR1 in breast cancer patients before and after treatment.

Feature/ Number of Patients (%)	Pre-Treatment sVEGFR1 Concentration (pg/mL)	Post-Treatment sVEGFR1 Concentration (pg/mL)	
	Median (Q1–Q3)	Median (Q1–Q3)	*p*-Values
BCS + Radiotherapy n = 70 (83%)	31.40 (22.27–87.12)	343.20 (218.80–392.10)	**0.0001**
Mastectomy n = 14 (17%)	27.09(19.60–76.27)	282.95 (255.60–362.90)	**0.0005**
Chemotherapy			
Anthracycline n = 30 (36%)	29.58 (22.27–76.27)	292.25 (217.90–363.90)	**0.0001**
Non-anthracycline n = 8 (9%)	51.98 (18.89–84.39)	264.40 (222.15–329.20)	**0.0133**
No n = 46 (55%)	32.09 (19.68–97.24)	354.00 (255.60–407.30)	**0.0001**
Endocrine therapy			
Tamoxifen n = 41 (49%)	30.00 (19.20–87.12)	345.32 (249.20–407.30)	**0.0001**
Inhibitor aromatase n = 19 (23%)	41.99 (19.60–97.24)	343.20 (194.10–381.80)	**0.0001**
Tamoxifen and inhibitor aromatase n = 7 (8%)	22.46 (15.25–37.81)	295.50 (262.10–448.10)	**0.0233**
Other type n = 3 (4%)	82.14 (79.80–131.29)	262.10 (133.70–404.40)	0.2482
No n = 14 (17%)	29.58 (22.27–83.31)	320.70 (233.50–386.50)	**0.0005**

sVEGFR1—soluble form of vascular endothelial growth factor receptor type 1; BCS—breast conserving surgery; significant differences are denoted by bold *p*-values.

**Table 4:** Levels of sVEGFR2 in breast cancer patients before and after treatment.

Feature/ Number of Patients (%)	Pre-Treatment sVEGFR2 Concentration (pg/mL)	Post-Treatment sVEGFR2 Concentration (pg/mL)	
	Median (Q1–Q3)	Median (Q1–Q3)	*p*-Values
BCS + Radiotherapy n = 70 (83%)	9275.74 (7418.77–11,955.8)	7397.15 (6800.0–8660.0)	**0.0001**
Mastectomy n = 14 (17%)	10,140.80 (8206.39–12,913.9)	7165.78 (6773.85–8770.0)	**0.0033**
Chemotherapy			
Anthracycline n = 30 (36%)	8751.85 (6680.40–11,092.93)	7327.40 (6316.18–8605.25)	**0.0176**
Non-anthracycline n = 8 (9%)	8773.58 (8287.31–11,612.07)	7113.08 (6817.50–8909.08)	0.0771
No n = 46 (55%)	9990.63 (7839.68–12,417.90)	7422.15 (6800.00–8759.71)	**0.0002**
Endocrine therapy			
Tamoxifen n = 41 (49%)	9868.01 (7740.15–12,417.90)	7458.00 (6825.00–8716.72)	**0.0018**
Inhibitor aromatase n = 19 (23%)	9581.91 (7582.57–12,426.22)	7400.00 (7061.47–8465.00)	**0.0059**
Tamoxifen and inhibitor aromatase n = 7 (8%)	9863.15 (8967.11–10,989.35)	8977.75 (6778.25–9392.83)	0.4497
Other type n = 3 (4%)	8216.21 (7839.68–9805.00)	6825.00 (6355.00–7310.00)	0.2482
No n = 14 (17%)	8770.23 (6559.79–12,113.50)	7089.92 (6109.41–8405.00)	0.1814

sVEGFR2—soluble form of vascular endothelial growth factor receptor type 2; BCS—breast conserving surgery; RTH—radiotherapy; significant differences are denoted by bold *p*-values.

**Table 5:** Univariate analysis (Cox regression) of pre- and post-treatment VEGF-A, sVEGFR1, sVEGFR2 concentrations in relation to disease-free survival (DFS) and overall survival (OS).

	OS		DFS	
Variables	HR (95% CI)	*p*-Values	HR (95% CI)	*p*-Values
VEGF-A pre-treatment Low High	1.4607 (0.4121–5.1774)	0.5572	0.9006 (0.5625–1.4422)	0.6632
VEGF-A post-treatment Low High	0.9960 (0.2883–3.4415)	0.9950	0.9412 (0.5878–1.5072)	0.8009
sVEGFR1 pre-treatment Low High	2.3581 (0.6065–9.1689)	0.2157	0.3104 (0.1898–0.5075)	**0.0001**
sVEGFR1 post-treatment Low High	1.6293 0.4597–5.7753)	0.4496	1.1525 (0.7201–1.8444)	0.5541
sVEGFR2 pre-treatment Low High	1.6702 (0.4667–5.9767)	0.4304	2.9558 (1.8071–4.8344)	**0.0001**
sVEGFR2 post-treatment Low High	4.4138 (0.9362–20.8098)	0.0606	0.9894 (0.6186–1.5825)	0.9646

VEGF-A—vascular endothelial growth factor A; sVEGFR1—soluble form of vascular endothelial growth factor receptor type 1; sVEGFR2—soluble form of vascular endothelial growth factor receptor type 2; significant differences are denoted by bold *p*-values; HR—hazard ratio; CI—confidence interval.

**Table 6:** Multivariate analysis (Cox regression) of pre- and post-treatment VEGF-A, sVEGFR1. sVEGFR2 concentrations in relation to disease-free survival (DFS) and overall survival (OS).

	OS		DFS	
Variables	HR (95% CI)	*p*-Values	HR (95% CI)	*p*-Values
VEGF-A pre-treatment Low High	2.0519 (0.5063–8.3163)	0.3141	0.6273 (0.3656–1.0764)	0.0905
VEGF-A post-treatment Low High	0.5997 (0.1345–2.6723)	0.5024	0.7312 (0.3999–1.3370)	0.3093
sVEGFR1 pre-treatment Low High	2.9007 (0.6533–12.8791)	0.1614	0.2170 (0.1204–0.3912)	**0.0001**
sVEGFR1 post-treatment Low High	3.0377 (0.7299–12.6419)	0.1267	1.1074 (0.6279–1.9531)	0.7243
sVEGFR2 pre-treatment Low High	2.1030 (0.5232–8.4531)	0.2949	4.2506 (2.3292–7.7571)	**0.0001**
sVEGFR2 post-treatment Low High	4.1921 (0.8591–20.4557)	0.0764	1.0415 (0.6114–1.7743)	0.8810

VEGF-A—vascular endothelial growth factor A; sVEGFR1—soluble form of vascular endothelial growth factor receptor type 1; sVEGFR2—soluble form of vascular endothelial growth factor receptor type 2; significant differences are denoted by bold *p*-values; HR—hazard ratio; CI—confidence interval.

**Table 7:** Linear regression models for disease-free survival predictors in breast cancer patients.

		Model 1	Model 2	Model 3	Model 4
VEGF-A pre-treatment	Beta *p*-value	0.1898 0.1576	0.2047 0.1288	0.2183 0.0995	0.2958 **0.0308**
VEGF-A post-treatment	Beta *p*-value	−0.0247 0.8580	0.0071 0.9590	0.0157 0.9090	−0.0506 0.7116
sVEGFR1 pre-treatment	Beta *p*-value	−0.1823 0.1696	−0.2218 0.1003	−0.2578 **0.0499**	−0.1425 0.2836
sVEGFR1 post-treatment	Beta *p*-value	−0.0541 0.6385	−0.0815 0.4901	−0.1149 0.3274	−0.0312 0.7950
sVEGFR2 pre-treatment	Beta *p*-value	−0.1335 0.3537	−0.1819 0.2181	−0.1600 0.2651	−0.0816 0.5839
sVEGFR2 post-treatment	Beta *p*-value	−0.0345 0.7741	−0.0252 0.8357	−0.0264 0.8257	0.0393 0.7471

Model 1—adjusted for age. Model 2 adjusted for age, BMI, parity, menopausal status. Model 3 adjusted for age, BMI, parity, menopausal status and smoking status. Model 4 adjusted for age. BMI, parity, menopausal status, smoking status, tumor stage, tumor diameters, intrinsic type, histological type, nodal involvement. VEGF-A –vascular endothelial growth factor A; sVEGFR1—soluble form of vascular endothelial growth factor receptor type 1; sVEGFR2—soluble form of vascular endothelial growth factor receptor type 2; significant differences are denoted by bold *p*-values.

## Data Availability

The data presented in this study are available on request from the corresponding author. The data are not publicly available due to privacy restrictions.
